# Epidemiological and Clinical Profile of Cleft Lip and/or Palate in Mbujimayi: A Cross‐Sectional Study

**DOI:** 10.1002/hsr2.73171

**Published:** 2026-09-01

**Authors:** Trésor Kabuya Kabamba, Eugène Mukeba Bamusua, Pascal Cimpaka Kabeya, Pascal Kayembe Shambuyi, Micheline Nyembo Epupwa, Didier Mubenga Katende, Séraphin Binene Katulondi, Henry‐Benjamin Tshimanga Kabeya, Jenny Nshimba Kabemba, Joël Manyonga, Joseph Luboya Kabila, Hubert Kabanga Ngandu, Roger Kamwema Shamuana, François Kabumba Kabumba

**Affiliations:** ^1^ Department of Surgery Université Officielle de Mbujimayi Mbujimayi Kasaï‐Oriental DR Congo; ^2^ Department of Pediatrics Université Officielle de Mbujimayi Mbujimayi Kasaï‐Oriental DR Congo; ^3^ Department of Exact Sciences Institut Supérieur Pédagogique Katanda Kasaï‐Oriental DR Congo; ^4^ Department of Public Health Institut des Techniques Médicales Ngandajika Lomami DR Congo; ^5^ Department of Internal Medicine Université Officielle de Mbujimayi Mbujimayi Kasaï‐Oriental DR Congo

**Keywords:** cleft lip and/or palate, clinical profile, epidemiology, Mbujimayi

## Abstract

**Background and Aim:**

Cleft lip and/or palate (CLP) is the most common craniofacial malformation worldwide, with significant geographic and socioeconomic disparities. In Mbujimayi, DRC, no prior data have been published on its prevalence or clinical presentation. This study aimed to determine the epidemiological and clinical profile of CLP in Mbujimayi.

**Methods:**

This was a cross‑sectional study conducted at the University Clinics of Mbujimayi between March 2023 and July 2025. A total of 54 patients with CLP were included. Ethical approval was obtained. Data were collected on demographic characteristics, anatomical types of CLP, and associated complications.

**Results:**

Unilateral cleft lip was the most frequent anatomical type, predominantly affecting the left side (48.1%). The mean age of patients was 5.5 years, with extremes ranging from 0.5 to 20 years. Feeding difficulties (40.7%), speech disorders, malnutrition, and social integration challenges (27.8%) were among the most common complications. A family history of CLP was reported in 13.0% of cases, and one associated malformation (cryptorchidism) was noted. The absence of a regional registry and reliance on free surgical campaigns limited the assessment of prevalence and postoperative outcomes.

**Conclusion:**

CLP is relatively common in Mbujimayi and is accompanied by significant clinical and psychosocial complications. Its management remains a major challenge in this resource‐limited setting. The establishment of a congenital malformation registry and structured follow‐up programs is essential to improve care and guide future research.

**Trial Registration:**

Not applicable.

**Abbreviations**
BCL: bilateral cleft lip
BCLP: bilateral cleft lip palate
LUCL: left unilateral cleft lip
LUCLP: left unilateral cleft lip and palate
RUCL: right unilateral cleft lip
RUCLP: right unilateral cleft lip and palate

## 1 Introduction

Cleft lip and/or palate (CLP) is among the most prevalent congenital malformations worldwide [1, 2]. Although its etiology remains incompletely understood, the prevailing hypothesis in the medical literature supports a multifactorial origin, implicating both genetic and environmental factors as potential contributors [3, 4, 5].

CLP develops during early pregnancy, typically between the sixth and eighth week of gestation. These anomalies result from a failure in the fusion of facial prominences. Their various clinical presentations are closely linked to the underlying embryological pathology, reflecting both the complexity and severity of the condition [6, 7].

CLP represents a significant public health concern due to its high incidence and the associated complications in affected children [8, 9]. Reported prevalence rates vary widely across countries and over time [10, 11]. In Europe, the prevalence of CLP is 1.6 per 1000 live births, whereas it is 0.44 per 1000 live births in African countries such as Ethiopia [12, 13].

Depending on its severity, CLP can lead to numerous complications for affected children, including dental anomalies, feeding difficulties, growth delays, facial dysmorphism, impaired self‐image, and social marginalization—all of which may significantly impact academic performance. These challenges also place considerable emotional and logistical burdens on parents [14, 15, 16].

Accurate estimation of the prevalence of CLP in the Democratic Republic of Congo (DRC) remains challenging due to the absence of a systematically maintained national registry. The lived experience of affected individuals is further complicated by societal stigma and a lack of adequate healthcare resources, both of which pose significant barriers to effective management and support for patients and their families.

This study aimed to characterize the epidemiological and clinical profile of CLP in the city of Mbujimayi.

## 2 Methods

### 2.1 Study Design and Framework

This was a cross‐sectional study conducted in the surgical department of the University Clinics of Mbujimayi, DRC, between March 2023 and July 2025.

The University Clinics of Mbujimayi are the largest surgical referral center in the city and represent the final level of specialized care, serving a population of 3.3 million inhabitants. They constitute the sole referral center for the management of CLP in the region. Patient recruitment was consecutive.

Patient selection was performed by two surgeons who confirmed the clinical diagnosis and verified eligibility according to predefined criteria.

All patients with CLP who presented to the University Clinics of Mbujimayi—whether through outpatient consultations, hospital admissions, referrals from peripheral health facilities, or identification during reconstructive surgery campaigns conducted during the study period—were included. These campaigns are organized exclusively at the University Clinics of Mbujimayi for the entire region, ensuring that patients with CLP are systematically referred or present spontaneously.

Patients were excluded if they had craniofacial malformations other than CLP, if they resided outside the catchment area of the University Clinics of Mbujimayi, or if their medical records lacked essential data for analysis.

In total, 54 patients were diagnosed and managed for CLP during the study period and constituted the study sample.

Patients with CLP received standardized care according to protocols proposed by Smile Train and validated by the department, covering evaluation, preoperative preparation, operative techniques, and postoperative follow‑up. Unilateral cleft lips were repaired using the Millard technique, while bilateral cleft lips were treated with a two‑stage reconstruction protocol. Palatal clefts were repaired by intravelar veloplasty and two‑flap palatoplasty, in accordance with the operative protocols applied during the campaigns. The operative timeline followed established principles, with lip repair performed during infancy and palate repair scheduled between 9 and 18 months depending on nutritional status and clinical eligibility. The procedures were performed free of charge by the Smile Train team in collaboration with local surgeons, which also enabled the training of a dedicated team for peri‑ and postoperative management and ensured continuity of care after the departure of external teams.

Infants with feeding difficulties received nutritional support, including positioning guidance, the use of specialized feeding bottles, and, when necessary, management of malnutrition in the pediatric department. Cases requiring specialized follow‑up (speech therapy, ENT, dentistry, and psychosocial support) were referred to the appropriate services when available.

Ethical approval for this study was obtained from the Ethics Committee of the Official University of Mbujimayi, in accordance with the principles outlined in the Declaration of Helsinki. Informed consent was obtained from all participants. For individuals under 18 years of age, consent was provided by a parent or legal guardian. All review procedures adhered to the ethical standards of the Mbujimayi University Clinic Research Commission.

### 2.2 Clinical Data

All participants included in this study underwent a standardized clinical examination conducted by trained surgical staff. The anamnesis focused on collecting sociodemographic and anthropometric data, identifying associated malformations and any family history of congenital anomalies, classifying the type of CLP, and documenting complications present at the time of consultation.

All patients underwent a complete clinical examination to screen for associated congenital anomalies. Systematic screening included the assessment of the extremities, abdominal wall, external genitalia, spine, and a cardio‑respiratory and neurological evaluation. Imaging was not performed routinely but was requested when clinical signs warranted further investigation. Complications were also diagnosed during clinical evaluation, including weight and/or mid‑upper arm circumference measurement, as well as ENT and respiratory assessment. A patient could present with one or several complications at the time of evaluation.

Socioeconomic status was assessed using a simplified household asset index derived from the Demographic and Health Surveys (DHS). This index classifies households into three socioeconomic levels—low (score 1–4), middle (score 5–7), and high (score 8–10)—based on household assets (radio, television, mobile phone, means of transport, housing materials, etc.) and access to drinking water, sanitation facilities, and electricity. In the present study, these three categories were used to ensure standardized classification comparable to national data.

Birth was considered preterm when it occurred between 22 and 37 completed weeks of gestation.

### 2.3 Statistical Analyses

Data were collected using an anonymized questionnaire, entered into an Excel spreadsheet, and analyzed with the Statistical Package for the Social Sciences (IBM SPSS, version 23.0). Categorical variables were summarized as frequencies and percentages, while continuous variables were expressed as means with standard deviations. Results were presented in tables and figures, as appropriate.

A theoretical estimation of the regional prevalence of CLP was performed to situate our findings within the ranges reported in the international literature. This estimation was based on demographic scenario modeling applied to the study period. During this period, the total number of live births in the Mbujimayi region was estimated at an annual mean of 112,725, with extreme values of 92,751 (high‑estimate scenario) and 140,699 (low‑estimate scenario), according to available regional demographic projections. Among the 54 included patients, only 29 were born between March 2023 and March 2025; when related to these two extreme birth estimates, a theoretical prevalence ranging from 0.21‰ to 0.31‰ was obtained, using the formula:

$$\mathrm{Prevalence}\;(‰)=\frac{\mathrm{number}\;\mathrm{of}\;\mathrm{cases}\;\mathrm{of}\;\mathrm{CLP}}{\mathrm{number}\;\mathrm{of}\;\mathrm{live}\;\mathrm{births}}\times 1000$$

This range does not reflect the actual observed prevalence but represents a theoretical estimate intended to allow comparison with other regions in the absence of exhaustive demographic data.

## 3 Results

### 3.1 Sociodemographic Characteristics of Patients

#### 3.1.1 Gender and Age

Of the 54 patients included in the study, 31 were male (57.4%), and 23 were female (42.6%). The mean age was 5.5 years. The most represented age group was infants under 1 year, accounting for 44.5% of cases (Table 1).

**Table 1:** Patient distribution by sex and age.

Variables	Value (*n* = 54)		%
Sex			
Male	31		57.4
Female	23		42.6
Age (years)			
< 1	24		44.5
1–5	14		25.9
6–10	6		11.1
11–15	4		7.4
≥ 16	6		11.1

*Note:* Sex ratio M/F: 1.4. Average age: 5.5 ± 4.4 years. Extremes: 0.5 and 20 years.

### 3.2 Other Sociodemographic Characteristics and Patient Background

The majority of patients originated from rural areas (92.6%) and belonged to low socioeconomic households (83.3%). The mean maternal age was 29.6 years. Birth weight was within the normal range in 55.5% of cases. A family history of CLP was reported in 13.0% of patients. Only one case of an associated malformation—cryptorchidism—was identified, representing 1.9% of the study population (Table 2).

**Table 2:** Other sociodemographic characteristics and perinatal characteristics of patients.

Variables	Value (*n* = 54)		%
Origin			
Rural environment	50		92.6
Urban environment	4		7.4
Socioeconomic level			
Down	45		83.3
Average	9		16.7
Mother's age (years)			
< 20	6		11.1
20–29	24		44.5
30–39	14		25.9
≥ 40	10		18.5
Gestational age			
Full‐term delivery	43		79.6
Premature birth	6		20.4
Birth weight (g)			
< 2500	11		20.4
2500–3999	30		55.5
≥ 4000	13		24.1
Family history of FLP			
No	47		87.0
Yes	7		13.0
Associated malformations			
No	53		98.1
Yes (cryptorchidism)	1		1.9

*Note:* Average age of mothers: 29.6 ± 8.2 years. Average birth weight: 3380 ± 790 g.

#### 3.2.1 Types of Cleft Lip and/or Palate

**Figure 1:**
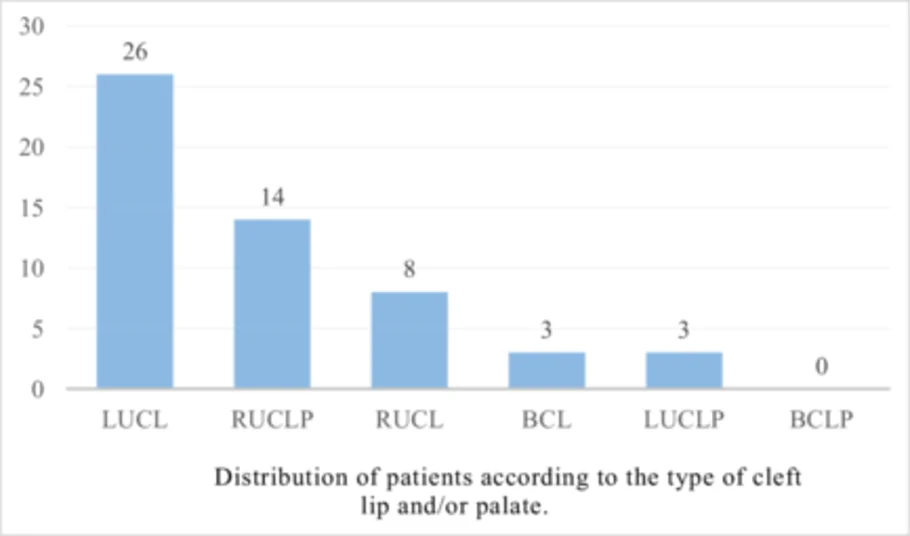
Distribution of patients according to the type of cleft lip and/or palate. BCL, bilateral cleft lip; BCLP, bilateral cleft lip and palate; LUCL, left unilateral cleft lip; LUCLP, left unilateral cleft lip and palate; RUCL, right unilateral cleft lip; RUCLP, right unilateral cleft lip and palate.

Unilateral cleft lip was the most frequent anatomical presentation, predominantly affecting the left side in 26 patients (48.1%). The second most common form was unilateral cleft lip and palate, with right‐sided involvement observed in 25.9% of cases (Figure 1).

#### 3.2.2 Complications of Cleft Lip and/or Palate

**Figure 2:**
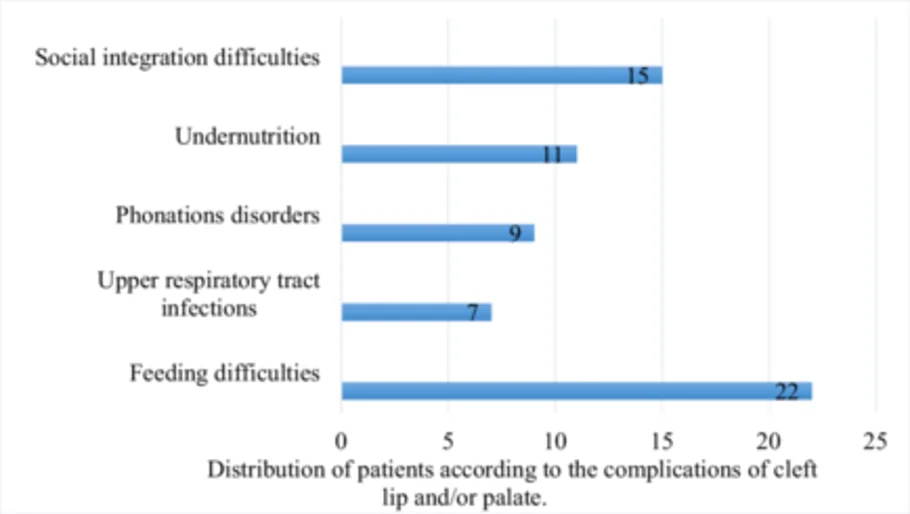
Distribution of patients according to the complications of cleft lip and/or palate.

These findings underscore the dual burden of CLP, affecting both physiological function and psychosocial well‐being. At the time of consultation, 22 patients (40.7%) reported experiencing feeding difficulties. Additionally, 15 parents or guardians (27.8%) expressed concerns regarding their child's social acceptance and inclusion (Figure 2).

A patient could present with one or several complications at the time of evaluation.

## 4 Discussion

CLP is the most common craniofacial malformation worldwide. However, its reported incidence varies considerably across studies, with notable racial and geographic disparities [17, 18]. Estimates range from 6 per 1000 to 1.37 per 1000 live births in the literature [19, 20, 21]. Higher prevalence rates have been documented in regions such as South Asia and South America, reaching approximately 1.47 per 1000 births [22, 23, 24]. These disparities may be attributed to differences in birth rates, study durations, and sample sizes across populations.

Due to the absence of a regional registry for congenital malformations, the exact prevalence of CLP in Mbujimayi remains difficult to determine. However, based on local birth rate estimates, the prevalence of CLP can be approximated at between 0.21 and 0.31 cases per 1000 live births.

The male predominance observed in this study aligns with findings from several international series, including those by Sundoro and Zhou [25, 26, 27]. The mean age of patients was 5.5 years, with a range from 0.5 to 20 years. Similar age distributions have been reported in other studies, typically ranging from 4 to 6 years [28, 29], although older ages have also been documented [30]. In our context, the relatively high age at the time of consultation highlights a critical issue: the limited financial capacity of families, who often lack access to insurance coverage or charitable support. This socioeconomic barrier to early care has also been noted by Vu et al. [31].

Beyond international comparisons, several contextual factors specific to Mbujimayi influence the clinical presentation and management of CLP. The health infrastructure remains limited, with specialized services concentrated at the University Clinics of Mbujimayi, leading to delays in diagnosis and treatment.

The predominance of patients from rural areas and low socioeconomic households reflects structural inequalities in access to healthcare in the DRC. Prenatal ultrasound and specialized services remain scarce in rural settings, and healthcare provision still largely relies on direct out‑of‑pocket payment by families. Although a national Universal Health Coverage program has recently been launched to subsidize maternal care, this initiative remains limited, is not operational in remote areas, and faces major financial and logistical challenges. Furthermore, elective abortion is legally restricted, and the predominantly Christian religious context discourages pregnancy termination, which contributes to the rarity of prenatal detection and the late presentation of children with CLP.

Local cultural practices also play an important role. In some families, CLP may be perceived as a sign of curse or transgression, resulting in concealment of the child or delayed care‑seeking. Furthermore, the low utilization of maternal and neonatal health services, combined with a high rate of home deliveries, contributes to underreporting of cases and late presentation.

These contextual elements partly explain the high proportion of older children at the time of first consultation and the difficulty in establishing a neonatal incidence that is representative of the general population.

In this series, the mean maternal age was 29.6 years. This finding is consistent with the study by Doray et al., which reported maternal ages of 28.8 and 29.2 years, depending on the anatomical subtype of CLP considered [32].

In this study, a family history of CLP was reported in 13.0% of patients, while only one case of an associated malformation—cryptorchidism—was identified (1.9%). These findings are comparable to those reported by Camus, who observed a family history of CLP in 13.6% of cases [33]. Similarly, De Berail noted that the prevalence of associated malformations in CLP patients tends to be lower, varies according to the type of cleft, and is predominantly urogenital in nature [34].

These findings suggest the involvement of both genetic and environmental factors in the pathogenesis of CLP, acting either independently or in combination. This multifactorial model may account for the recurrence of CLP in certain families, as previously affected individuals may share underlying genetic susceptibilities or be exposed to similar environmental conditions.

Unilateral cleft lip was the most frequent anatomical presentation, with predominant involvement of the left side in 26 patients (48.1%). This pattern has been similarly reported by several authors worldwide [32, 35]. Sundoro et al. observed a comparable distribution, with 46.4% of cases involving left‐sided unilateral cleft lip in their series [25]. Despite advances in embryopathological research, the predominance of left‐sided clefts remains poorly understood and lacks a definitive explanation.

Far from being a benign condition, CLP is associated with a range of complications, some of which may be severe or even life‐threatening. In the present study, patients presented with feeding difficulties, speech disorders, malnutrition, respiratory infections, and challenges related to social integration. These complications are well‐documented in the literature [36, 37, 38]. In Mbujimayi, the occurrence of CLP is often perceived as a source of stigma, with significant psychological repercussions for parents and caregivers. This perception is consistent with findings from a study conducted in India by Kumar et al., which highlighted the emotional burden and social isolation experienced by families affected by CLP [39].

In light of these findings, it is imperative to underscore the importance of regular follow‐up and early surgical intervention for patients with CLP. Such management is essential to prevent complications and improve long‐term outcomes. However, in resource‐limited settings such as Mbujimayi, access to timely and comprehensive care remains a major challenge, highlighting the need for targeted health policies and support systems.

In this perspective, several priority actions can be considered: the establishment of a regional registry for the surveillance of congenital malformations, which would allow systematic case reporting and prevalence estimation; the strengthening of local surgical capacities through continuous training programs to decentralize patient management and facilitate timely access to care; and the improvement of social services to ensure appropriate psychosocial and nutritional support for patients and their families, thereby enhancing community understanding and adherence.

## 5 Limitations of the Study

Although this study has the merit of being the first to document CLP in the city of Mbujimayi, it presents several limitations, notably:

– The inability to determine the true prevalence of CLP in Mbujimayi due to the wide variation in patient age at the time of consultation, as well as the absence of risk‑factor identification, which was not possible in the absence of a regional congenital malformation surveillance registry. The implementation of such a registry has been proposed to generate reliable data for future studies.
– Furthermore, because patients are generally managed during free cleft repair campaigns, it was difficult to assess long‑term postoperative outcomes, and the frequency of complications may be underestimated. Associated malformations may also be underdiagnosed due to the lack of systematic imaging. These limitations nonetheless provide valuable perspectives for future research in Mbujimayi.

## 6 Conclusion

This study provides the first description of the clinical characteristics of patients with CLP managed in Mbujimayi. The findings primarily reflect local patterns of access to care, characterized by late presentation and frequent complications such as feeding difficulties and malnutrition.

Although the collected data do not allow estimation of the true prevalence in the general population, they highlight persistent challenges related to early diagnosis, continuity of follow‑up, and access to specialized care. These observations underscore the need to strengthen maternal and neonatal health infrastructure, improve community awareness, and develop more structured and accessible management programs.

## Author Contributions

**Trésor Kabuya Kabamba:** conceptualization, writing – original draft, writing – review and editing, project administration. **Eugène Mukeba Bamusua:** writing – original draft, writing – review and editing. **Pascal Cimpaka Kabeya:** validation, methodology, resources. **Pascal Kayembe Shambuyi:** investigation, data curation. **Micheline Nyembo Epupwa:** formal analysis, software. **Didier Mubenga Katende:** software, formal analysis. **Séraphin Binene Katulondi:** writing – review and editing, supervision. **Henry‐Benjamin Tshimanga Kabeya:** methodology, validation, resources. **Jenny Nshimba Kabemba:** data curation, investigation. **Joël Manyonga:** data curation, investigation. **Joseph Luboya Kabila:** writing – review and editing, visualization. **Hubert Kabanga Ngandu:** software, formal analysis. **Roger Kamwema Shamuana:** visualization, writing – review and editing. **François Kabumba Kabumba:** writing – review and editing, supervision.

## Funding

The authors have nothing to report.

## Conflicts of Interest

The authors declare no conflicts of interest.

## Transparency Statement

The lead author Trésor Kabuya Kabamba affirms that this manuscript is an honest, accurate, and transparent account of the study being reported; that no important aspects of the study have been omitted; and that any discrepancies from the study as planned (and, if relevant, registered) have been explained.

## Data Availability

The data that support the findings of this study are available from the corresponding author upon reasonable request.
